# Early development of infants with neurofibromatosis type 1: a case series

**DOI:** 10.1186/s13229-017-0178-0

**Published:** 2017-11-23

**Authors:** Anna May Kolesnik, Emily Jane Harrison Jones, Shruti Garg, Jonathan Green, Tony Charman, Mark Henry Johnson, the EDEN-BASIS Team+, Simon Baron-Cohen, Jannath Begum, Patric Bolton, Celeste Cheung, Leila Dafner, Kim Davies, Mayada Elsabbagh, Janice Fernandes, Laurel Fish, Isobel Gammer, Marian Greensmith, Teea Gliga, Sarah Kalwarowsky, Michelle Liew, Greg Pasco, Andrew Pickles, Helena Ribeiro, Erica Salomone, Chloe Taylor, Leslie Tucker, Sam Wass, Emma Burkitt-Wright, D. Gareth Evans, Grace Vassallo, Judith Eelloo, Siobhan West, Elizabeth Howard, Eileen Hupton, Sue Huson, Lauren Lewis, Karen Tricker, Angus Dobbie, Ruth Drimer, Saghira Malik Sharif, Diane Baralle, Carolyn Redman, Saba Sharif, Carolyn Symth, Wayne Lam, Alyson Bradbury, Neil Harrower, Oliver Quarrell, Helen Bethell, Rachel Jones, Susan Musson, Catherine Prem, Miranda Splitt, Karen Horridge, Christine Steiger, Carly Jim

**Affiliations:** 1 Centre for Brain and Cognitive Development and Department of Psychology, Birkbeck, University of London, Malet Street, London, WC1E 7HX UK; 2 Neuroscience & Experimental Psychology, Manchester Academic Health Science Centre, University of Manchester and Royal Manchester Children’s Hospital, Central Manchester University Hospitals NHS Foundation, Manchester, UK; 3 Department of Psychology, Institute of Psychiatry, Psychology & Neuroscience, King’s College London, London, UK

**Keywords:** NF1, Infant, Translational neurodevelopment, Autism, Prospective longitudinal, Adaptive functioning, Cognition, Sensory processing, Social engagement, Development

## Abstract

**Background:**

Prospective studies of infants at familial risk for autism spectrum disorder (ASD) have yielded insights into the earliest signs of the disorder but represent heterogeneous samples of unclear aetiology. Complementing this approach by studying cohorts of infants with monogenic syndromes associated with high rates of ASD offers the opportunity to elucidate the factors that lead to ASD.

**Methods:**

We present the first report from a prospective study of ten 10-month-old infants with neurofibromatosis type 1 (NF1), a monogenic disorder with high prevalence of ASD or ASD symptomatology. We compared data from infants with NF1 to a large cohort of infants at familial risk for ASD, separated by outcome at age 3 of ASD (*n* = 34), atypical development (*n* = 44), or typical development (*n* = 89), and low-risk controls (*n* = 75). Domains assessed at 10 months by parent report and examiner observation include cognitive and adaptive function, sensory processing, social engagement, and temperament.

**Results:**

Infants with NF1 showed striking impairments in motor functioning relative to low-risk infants; this pattern was seen in infants with later ASD from the familial cohort (HR-ASD). Both infants with NF1 and the HR-ASD group showed communication delays relative to low-risk infants.

**Conclusions:**

Ten-month-old infants with NF1 show a range of developmental difficulties that were particularly striking in motor and communication domains. As with HR-ASD infants, social skills at this age were not notably impaired. This is some of the first information on early neurodevelopment in NF1. Strong inferences are limited by the sample size, but the findings suggest implications for early comparative developmental science and highlight motor functioning as an important domain to inform the development of relevant animal models. The findings have clinical implications in indicating an important focus for early surveillance and remediation in this early diagnosed genetic disorder.

**Electronic supplementary material:**

The online version of this article (10.1186/s13229-017-0178-0) contains supplementary material, which is available to authorized users.

## Background

Autism spectrum disorder (ASD) is a neurodevelopmental disorder that affects social communication and flexible behaviour in up to 1.5% of the population [1]. Identifying causal paths that link genetic and environmental risk factors to later behaviour is a critical target for the field, because of the potential to yield new routes to intervention. Symptoms of ASD likely emerge through a complex developmental cascade of interactions between genetics, the brain, cognition, behaviour, and the child’s interaction with their environment [2]. ASD can be reliably diagnosed by ages 2 to 3[3], and risk factors act predominately prenatally [4, 5]. Thus, a focus on early brain development is critical to differentiating putative causal paths from compensatory, adaptive, or secondary cascading effects of early symptoms [6]. Linking those causal paths to specific neurobiological systems is critical to developing new pharmacological treatments to complement existing behavioural therapies [7].

Prospective longitudinal studies of infants with older siblings with ASD have yielded significant insights into its earliest features in this group [2]. In the first year of life, behavioural differences in infants with later ASD are difficult to detect and may be most common in sensory and motor functioning [8, 9]. For example, 6-month-old infants with later ASD are more likely to show poor head control [10] and infants at familial risk as a group show poor postural control [11] and more limited reaching and grasping skills [12]. In contrast, social communication appears relatively typical in the first 6 months [8, 13], although neurocognitive measures reveal subtle vulnerabilities in social engagement [14], response to eye gaze [15], and declining interest in eyes [16]. By the end of the first year, clear differences are present in a range of ASD-related behavioural phenotypes, including poorer language and communication skills [8], reduced joint attention [17], diminishing social interest [13], and the emergence of unusual interests in objects [18]. Measures like the Autism Observational Scale for Infants begin to show reasonable sensitivity to ASD outcome at this time [19, 20], although diagnosis is still difficult until the second or third year of life [3]. Thus, it appears that clear delays in ASD-relevant domains emerge over the first year of life but may be preceded by alterations in early brain development that affect lower-level sensorimotor systems.

The unclear aetiology of ASD in infants at polygenic familial risk makes it difficult to translate such insights to animal models, which are mainly based on single gene knock-out approaches. This in turn limits our ability to tie insights to particular neurobiological systems or pathways and to generate new pharmacological treatment strategies. A complementary way to study the emergence of ASD that may facilitate translational insights is to test infants with defined genetic syndromes associated with a heightened incidence of ASD. Examples of conditions that can be potentially identified in early development, carry a high risk of ASD, and have been successfully modelled in animals include fragile X syndrome, tuberous sclerosis complex, and neurofibromatosis type 1 (NF1). One challenge to this approach is that these conditions are rare. Identified genetic syndromes only account for a small proportion of cases of ASD, and thus, the generalisability of mechanisms observed in particular disorders remain unclear [21, 22]. Thus, the optimal strategy may be to establish which antecedent biomarkers observed in studies of infants at familial risk are also present in infants with genetic syndromes. In this way, we can identify generalised causal paths that are likely widely applicable but that can also be more carefully probed at the molecular and neurobiological levels.

Prospective studies of infants with NF1 provide an important complementary approach in this context. In contrast to other monogenic syndromes, NF1 is not complicated by severe intellectual disability or seizures. NF1 is the most common autosomal-dominant single-gene condition associated with increased risk for neurodevelopmental disorders, with birth incidence of 1:2700 [23]. Fifty percent of the cases are inherited, while the rest are de novo cases due to spontaneous mutation of the NF1 gene located on chromosome 17q11.2, which encodes for the protein neurofibromin. Although well known for its cutaneous manifestations, the main challenges experienced by people with NF1 are cognitive, social, and behavioural. The overall IQ is in the low-average range, although specific learning impairments are common [24]. There is a high prevalence of ASD in NF1, with rates of 25% full ASD and 20% with partial ASD symptoms in the pediatric NF1 population [25]. The phenotypic profile of ASD in NF1 is also broadly similar to idiopathic ASD [26], making insights from NF1 more likely to generalise to understanding ASD more broadly.

The downstream molecular consequences of impaired NF1 function have been well characterised in NF1 knockout mouse models [27]. Impaired disinhibition of the Ras/MAP kinase pathway leads to changes in synaptic proteins, GABA/glutamate disequilibrium, and impairments in synaptic function [28]. Further, abnormalities in cyclic AMP and dopamine homeostasis underlie the attention system abnormalities in NF1 [29]. The social learning and attention impairments characteristic of ASD have been recapitulated in NF1 knock-outs [28, 30]. Targeted treatments such as lovastatin [31] and lamotrigine [32] reverse the NF1-associated cognitive impairments in knock-out models, but translational clinical trials in humans have so far had mixed results [33]. A major impediment for clinical trials is the lack of sensitive outcome measures on which to target treatment and a poor understanding of neurodevelopmental trajectories in children with NF1. Longitudinal studies mapping the developmental trajectories of children with NF1 will allow inferences about causal mechanisms as well as identify candidate biomarkers for future intervention studies. Treatments targeted in the prodromal period (before behavioural symptoms of ASD and ADHD emerge) could prevent or ameliorate the later emergence of symptoms [34].

Diagnosis of NF1 is made on clinical assessment using the National Institute of Health clinical consensus criteria. Since 50% of the cases are inherited, NF1 can be diagnosed in infancy using cord blood mutation testing or on clinical assessment. The comparison of developmental levels between infants with NF1 and infants with older siblings with ASD (defined on familial risk status) is thus putatively less confounded by ascertainment bias than for other comparable syndromes. Unless there are parental concerns, most infants with NF1 do not receive routine developmental monitoring. To our knowledge, this is the first study to report the developmental profile of NF1 in infancy.

### Present study

To understand early developmental profiles and the emergence of behaviours related to ASD in infants with NF1, we recently launched a prospective longitudinal study of this population. Our projected group size will be 30 infants with data on a rich battery of behavioural and neurocognitive measures at 5, 10, 14, 24, and 36 months; this report represents the first ten infants with data at 10 months enrolled in the study. This is a particularly interesting age, because it appears to mark the beginning of the emergence of behavioural signs of ASD in infants at familial risk [2]. The present study had two goals: first, to determine what developmental areas might be affected by the NF1 mutation in early development, and second, to examine whether infants with NF1 show profiles that resemble those of infants who develop ASD through other risk pathways. To do this, we compared the developmental profiles of infants with NF1 to those of a larger group of infants at high familial risk for ASD with different developmental outcomes at age 3 (typical development, ASD, or other atypical developmental profiles), in addition to a sample of low-risk typically developing infants. Taken together, our study provides the first evidence of the profile of developmental difficulties in infants with NF1 and their similarities and differences to profiles observed in infants at familial risk for ASD.

## Methods

### Participants

Participants described in this case series include ten 10-month-old children (4 male; 6 female) with an NF1 diagnosis through the Early Development in Neurofibromatosis Type 1 (EDEN) research project. NF1 is a rare disorder, with a birth incidence of approximately one in 2700 births in the UK per year [37]. Thus, our recruitment methods aimed to maximise the representativeness of our sample within the context of the rarity of the disorder. Participants were recruited via local and regional genetic centers (Manchester, Leeds, Newcastle, Southampton) and via advertisements placed in the NF charities’ social media webpages. The study has R&D approval for recruitment across all specialist genetic centers across the UK. The study information was offered to eligible participants at routine clinical appointments. Within the general population, NF1 is approximately 50% familial and 50% sporadic [35].Our sample consists primarily of familial cases (8/10) because they are typically identified earlier in development through cord blood testing. Of note, our previous behavioural phenotyping studies have shown no differences between familial and de novo cases [26].

All of the participants who had inherited NF1 were confirmed via molecular testing of cord blood samples (*n* = 8) or clinical diagnosis based on NIH consensus criteria (*n* = 2) [36]. Six of the infants had at least one older sibling. Behavioural and cognitive profiles of these infants were compared to a large corpus of data from the British Autism Study of Infant Siblings (BASIS, phases 1 and 2; http://basisnetwork.org). These children either had a family history of ASD and went onto receive a diagnosis at 36 months (HR-ASD, *n* = 34, 8F), had other indications of atypical development (such as high scores on ASD-related symptom measures or poor cognitive development; HR-Atyp, *n* = 43, 20F), or were typically developing (HR-no ASD, *n* = 89, 44F); or were ‘low-risk’ controls, i.e. had no family history of ASD (LR, *n* = 72, 37F). ASD diagnoses were made at age 3 years for research purposes only through expert clinical judgment based on all available clinical and behavioural assessments (including gold-standard ADOS and ADI measures) collected at 24 and 36 months (for details, see Additional file 1). The study was approved by the National Research Ethics Service London Central Ethical Committee and conducted in accordance with the Declaration of Helsinki (1964). As the study is ongoing, it is not yet possible to estimate how many of the children with NF1 will go on to receive an ASD diagnosis.

### Procedures

Informed consent was obtained from all families. All assessments took place at the Centre for Brain and Cognitive Development, Birkbeck, London. Behavioural measures described below were administered as part of a more extensive experimental protocol; for summary, see Additional file 1: Table S1.

#### Cognitive and adaptive skills

Cognitive ability was assessed through the Mullen Scales of Early Learning (M) administered according to the manual [37]. This is an observational measure that assesses gross and fine motor skills, expressive and receptive language, and visual reception. We report *t* scores per subdomain (*M* = 50, *SD* = 10), based on the US norms. Adaptive skills were assessed using the Vineland Adaptive Behavior Scale Parent Survey (VABS; [38]) form, a parent-report questionnaire that assesses socialisation, communication, motor behaviour, and daily living skills. We report standard scores per domain (*M* = 100, *SD* = 15) based on the US norms.

#### ASD symptoms

The Autism Observation Scale for Infants (AOSI; [39, 40]) is a 19-item interactive play schedule and was administered to five of the infants. It is designed to monitor early signs of ASD and measure aspects of visual attention, social communication, and development of sensory and motor skills. Absence/presence of behaviours is rated 0–3, where 0 signifies normal function, and higher values suggests increasing deviation from the normal behaviour expected at the age of assessment. Total scores range from 0–50 [19, 41].

#### Temperament

Parents completed the Infant Behavior Questionnaire (IBQ [42]), a parent-report measure that comprises 14 subscales grouped into three overarching factors labelled Surgency (the child’s tendency to show excitement, positive affect, and approach), Negative Affect (the child’s tendency to cry, be avoidant, or otherwise fussy), and Effortful Control (the child’s ability to regulate their mood and behaviour) [42, 43]. Parents of infants at high familial risk for ASD completed the original form of the IBQ-R [44]; parents of infants with NF1 completed the short form [42]. For comparability between cohorts, we rescored the long form of the IBQ-R by only selecting items also included on the short form.

#### Sensory processing

Parents completed the Infant/Toddler Sensory Profile (ITSP [45]). This questionnaire produces scores in four quadrants that reflect the child’s responsiveness to different types of sensory experiences. Sensation Seeking (generating higher sensory input for oneself) and Low Registration (noticing fewer sensory cues) encompass high-threshold responses. Sensory Sensitivity and Sensation Avoiding reflect low-threshold responses and are combined into a low-threshold quadrant score (detecting more sensory input than others, with negative affect and low self-regulation). Further, the scale includes five sensory processing scores for different processing systems (e.g. visual, auditory, tactile). Norms are available, and score ranges for typical performance or probable or definite differences are provided per subscale.

#### Examiner-rated behaviour

Levels of social engagement (SE) were examined through consensus coding of six different aspects of infant behaviour, including social affect, temperament, and vocalising; researchers used a 7-point Likert Scale at the end of the testing day ([46]; see Additional file 1).

#### Analytic strategy

We first present case vignettes for each infant. For measures with available norms (Mullen, VABS, and ITSP), we interpret scores as below average (< − 1SD) or low (< − 2SD) based on common convention using terminology specific to each measure. Sensory behaviours measured through the ITSP were rated as either probably more/less than others if one standard deviation away from the mean and definitely more/less than others when the score is two standard deviations away; note that cut-off scores vary per subdomain for this measure [45]. We also report qualitative summaries of non-standardised measures (IBQ, SE). However, it is important to note that all measures were administered in a research, not clinical context, and thus should not be considered diagnostic.

Second, we compared the performance of infants with NF1 at a group level with our other cohorts (HR-TD, HR-Atyp, HR-ASD, and LR) using ANCOVA with age as a covariate.

## Results

### Case summaries

Acronyms are used to identify the source of information provided about their level of cognitive/sensory development (e.g. IBQ, VABS). Descriptive data and scores are presented in Additional file 1: Tables S2, S3, S4, S5, and S6.

#### Case 1: male, diagnosis of NF1, at 10 months

Significant delays in gross motor skills (M, VABS), for example, he was not able to move from sitting to hands and knees, roll over, or pull up from supine to a sitting position. Fine motor abilities were below average for the age group, as he demonstrated a partial pincer grasp but could not take blocks in/out of a container or bang them together (M). Cognitive skills were within the average range (M). Infant demonstrated object permanence, appropriate use of objects, and understanding inhibitory commands. Expressive language was below average and consisted of voluntary babbling and consonant sounds, with no first words or jabbering with inflection (M); receptive language was adequate, but he had difficulty understanding verbal requests and questions from the examiner (M). Broader communication skills were moderately low (VABS). Social skills were a relative strength and judged at age-expected levels (VABS). Examiners reported moderate levels of eye contact and attentiveness, but relatively low levels of shared affect (SE). He showed altered sensory processing across all domains relative to age-appropriate norms (particularly auditory, vestibular, and oral processing); this mainly reflected more registration of sensory stimuli and a low threshold for noticing sensory changes (ITSP). By parent report of temperament, surgency, negative affect, and effortful control were all within one standard deviation of average values in the low-risk sample (IBQ).

#### Case 2: male, diagnosis of NF1, at 11 months

Gross motor skills were within the normal range by examiner observation (M), and he had strong fine motor skills (M); he had mastered balance and control of the upper/lower extremities and was gaining upright mobility. However, his overall adaptive motor skills were rated lower than average by parent report (e.g. sitting and crawling behaviours occurred lower relative to the age-matched population norm). Cognitive skills were a relative strength—he achieved object permanence and demonstrated early spatial awareness and visual memory (M). Communication skills were relatively poor, specifically in receptive language; he was able to understand simple verbal input (e.g. response to own name or familiar names/words) but failed to give a toy in response to a request and a gesture or identify an object after hearing it named (M). Expressive language was in the average range, marked by presence of first word, as well as communication of intentions through jargon combined with gestures. By parent report, his everyday communication behaviour was relatively strong, as were his social skills (e.g. responding to parent with vocalisation, engaging in games of ‘peek-a-boo’; VABS). Examiners reported frequency of eye contact, shared affect, and social responsiveness (SE). Temperamentally, his surgency and negative affect were within the normal range (though he showed high activity levels in the lab SE), but he showed relatively lower levels of effortful control than low-risk infants (IBQ). Sensory processing was considered typical for most domains, though he showed probably altered auditory processing and definitely less sensation seeking than other infants (ITSP).

#### Case 3: male, diagnosis of NF1, at 11 months (*)

Gross motor skills were very low (unable to sit independently), and fine motor skills were below average (M): he was able to grasp and manipulate objects, as well as display a partial pincer grasp, but was not able to use both hands together when playing with an object or turn pages in a book. Parent report suggests his use of motor skills in everyday life was adequate but gross motor skills were poorer than fine motor skills (VABS). He had marked difficulty in controlling motor behaviour, although no atypical motor/sensory behaviours specifically related to ASD were noted (AOSI). Cognitive skills were in the low-average range (M); he showed object permanence and began associating objects with functions but was unable to open/close a book or pay attention to pictures. Expressive language skills were very low; he did not babble or produce consonant sounds during the assessment (M). Receptive language was also below average; he was able to give a toy on request, but only when it was accompanied by a gesture, and was not able to understand simple questions from the experimenter (M). During the AOSI, he showed no social babbling. However, by parent report, his communication skills at home were adequate (VABS) and his social skills were strong (VABS). During the day, the child was rated as moderately socially responsive, with moderate eye contact and shared affect (SE), but he showed clearly atypical eye contact, reciprocal smiling, and social interest when probed (AOSI). Temperament was parent-rated as comparable to other groups for surgency and negative affect, but effortful control was lower than in low-risk controls (> − 1SD) (IBQ); examiners also rated him as moderately negative and quite active (SE). Sensory ratings indicated altered visual processing, and definitely ‘less’ low registration (meaning he was less likely to fail to notice sensory stimuli in the environment).

#### Case 4: male, diagnosis of NF1, at 12 months

Gross motor skills were very low for his age group (M, VABS). He was unable to pull up on furniture or get into a sitting position from hands and knees (M). Fine motor abilities were age-appropriate—he was able to use both hands to manipulate an object and displayed finer coordination of movement (M). Cognitive scores were a relative strength; he obtained object permanence and early spatial awareness and could associate an object and its function (M). Expressive language was very low—he was unable to voluntarily babble or produce any consonant sounds (M). He also showed below-average receptive language ability and was unable to identify an object or respond to a verbal request (M). By parent report, his communication skills were just in the average range. Daily living and social skills were adequate (VABS); examiners also rated his eye contact, affect, and social responsiveness as frequency (SE). Surgency and effortful control were within one standard deviation of the typical range (IBQ), and examiners rated his temperament as very positive (SE); however, by parent report, he showed elevated negativity relative to low-risk controls (IBQ). Sensory sensitivity was within the typical range for all scores, apart from probably altered sensitivity to vestibular sensations (items include needs support for sitting, enjoying physical play, and resists having head tipped back).

#### Case 5: female, diagnosis of NF1, at 9 months (*)

Gross motor skills were below average in the lab (M) and low in everyday contexts (VABS). Fine motor skills were below average; she was able to use a partial pincer grip, but not both hands together, or turn pages in a book sequentially (M). During the AOSI, she showed marked difficulty controlling motor behaviour, as well as atypical sensory and motor behaviours (AOSI). Cognitive skills were very delayed, with partial object tracking, no object permanence, and failure to associate objects with functions (M). However, by parent report, her daily living skills were typical (VABS). Communication skills were delayed (VABS); during testing, her receptive language skills were rated as below average, while expressive language was a relative strength and age-appropriate, with voluntary babbling and production of several words (M). Everyday social skills were below average (VABS); however, during the testing day, the child maintained relatively frequent eye contact and shared affect with the experimenter (SE) and showed good social skills on the AOSI. Temperamentally, her surgency (e.g. expression of pleasure) and negativity were within expected levels but effortful control was rated lower than low-risk controls (< − 1SD; IBQ). The child showed pervasive atypicalities in the sensory domain, with atypical auditory, visual, tactile, and oral sensory behaviours; these were likely related to more low registration (reduced attention towards environmental cues; ITSP).

#### Case 6: female, diagnosis of NF1, at 10 months

Gross motor abilities were at floor level in the lab (M), equivalent to a 3-month-old (M). Child was able to bear weight on forearms and hold her head steady in a supported seated position but was not able to roll over or grasp fingers and pull up from a supine into a sitting position. However, she performed at an ‘above average’ level on fine motor skills (M); adaptive fine motor skills were low but in the average range (VABS), showing evidence of the development of more precise coordination of movement. Visual reception skills were age-appropriate, for example, she achieved object permanence and was able to associate objects with functions (M). Expressive language was within the average range; she produced varied and controlled vocalisations and was able to say one recognisable word (M); receptive language skills were below average (M). Parent ratings also indicated moderately low communication abilities (VABS) but relatively strong social skills. During the testing day, she showed frequent eye contact, moderate shared affect, and social responsiveness. Temperamentally, she was rated during the testing day as showing a highly positive temperament though with low attentiveness (SE), and by parent report, surgency, negativity, and effortful control scores were within the average range of control groups (IBQ). Finally, she showed no overt atypicalities in sensory behaviours across all domains (ITSP).

#### Case 7: female, diagnosis of NF1, at 10 months (*)

Gross motor skills and adaptive motor behaviours were below average for the age range, but fine motor skills were typical (M, VABS) and she showed no motor atypicalities on the AOSI. She had good cognitive ability, including object permanence, early spatial awareness and visual memory (M). Language skills were poor, with low expressive language; she showed voluntary babbling and production of consonants but was unable to vocalise two-syllable sounds or produce first words as expected for the age group (M). She showed very poor receptive language (the 5-month level); she did not respond to her name or understand simple verbal input. Adaptive communication skills were considered strong by parent report (VABS). Social skills in everyday contexts were also typical by parent report (VABS); during testing, she showed frequent eye contact, moderate shared affect, and social responsiveness but became distressed when without her parents. She showed some evidence of diminished social responsiveness to an unfamiliar examiner when promoted (A). Temperamentally, surgency and effortful control were low (< − 1SD) and negative affect was high (> + 2SD) relative to typical controls, although was rated as relatively active and attentive (SE). Sensory responses were altered for auditory and visual domains, likely because she is more likely to notice less sensory cues from the immediate environment (ITSP).

#### Case 8: female, diagnosis of NF1, at 10 months (*)

Gross motor functioning was poor; she was unable to pull herself to stand or move from sitting position to hands and knees (M); this was accompanied by atypical motor control and behaviours, such as hand waving (AOSI). She also showed poor fine motor skills and was unable to manipulate objects or demonstrate a pincer grasp (M); adaptive motor function was also rated low by parents (VABS). Cognitive skills were below average (although an area of relative strength), with partial object permanence (M), and poor disengagement (AOSI). Language skills were also significantly delayed in both expressive and receptive domains (M) and in everyday contexts (VABS). She was unable to voluntarily babble or produce consonant sounds and showed absent responses to sound or voice/face of the experimenter (M). Social skills in an everyday context were also delayed (VABS). In the lab, she showed moderate to poor eye contact but poor shared affect and social responsiveness (AOSI; SE). Temperamentally, her levels of surgency were relatively low (> − 1SD), as was her effortful control (IBQ). During the testing day, she showed low attentiveness and activity level (SE). Parent-rated reports indicated atypical sensory behaviours across auditory and visual/vestibular and oral sensory domains, mainly related to higher levels of failing to notice sensory stimuli as well as a higher likelihood to have a low threshold for distress reactions (ITSP).

#### Case 9: female, diagnosis of NF1, at 11 months

Gross motor function was poor; she could sit independently but was not able to walk with one hand held or stand up independently (M). Fine motor skills were typical, including using both hands together and coordinated movements (M); composite motor skills in an everyday context were low to average (VABS). Cognitive skills were below average—she was able to show object permanence and appropriate use of objects but failed to pay attention to pictures shown by experimenter (M). Expressive language skills were very low, with no voluntary babbling or consonant sounds (M). Receptive language was just below average, including giving a toy in response to a verbal request and understanding actions (i.e. waving goodbye or clapping). Overall adaptive communication was rated as adequate (VABS). Social skills were also rated as adequate (VABS), and during the day, she showed frequent eye contact, shared affect, and generally positive temperament (SE). Temperamentally, she showed low surgency and negative affect (< 1SD) relative to low-risk controls; effortful control was high (> 1SD). In the sensory domain, she showed a broadly typical profile, with altered visual processing, probably less likelihood of failing to notice sensory stimuli and probably more sensation seeking (ITSP).

#### Case 10: female, diagnosis of NF1, at 11 months (*)

Gross motor abilities were low, though she was able to sit independently and turn to reach a toy placed on the side (M). Fine motor skills were also below average, including a partial pincer grasp and object manipulation; however, she was unable to use both hands together or turn pages in a book (M). Adaptive motor skills at home were in the normal range by parent report (VABS), but she showed atypical motor control and behaviours during interaction with an examiner (AOSI). Her visual reception skills were low-average, though she understood object permanence and simple problem solving (M). Expressive language was below average; she is able to babble voluntarily but could not produce consonant sounds and first words or engage in a gesture/language game such as ‘peek-a-boo’ (M). She was functioning at a very low range on receptive language; she did not respond to own name or understand simple verbal input (M). Adaptive communication skills were moderately low by parent report (VABS). Social skills were within the typical range; in the lab, she showed relatively frequent eye contact, but limited shared affect and social responsiveness and social babbling (SE, AOSI). Temperamentally, she showed relatively high levels of negative affect (> 2SD), low effortful control (< 2SD), and high surgency (> 1SD) (IBQ). Sensory behaviours were within the typical range (ITSP).

### Group analysis

#### Motor skills

**Fig. 1:**
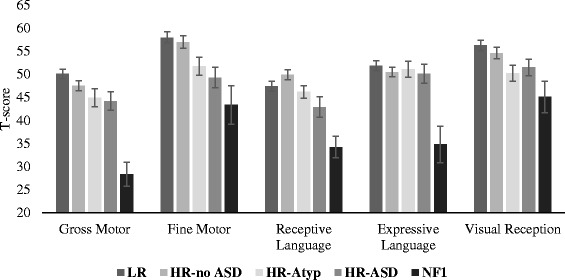
Average standard scores for the subscales of the Mullen Scale of Early Learning from groups of infants with LR (low familial risk of ASD), HR-no ASD (high familial risk with later typical development), HR-Atyp (high familial risk with other atypical developmental profiles), HR-ASD (high familial risk with later ASD outcome), and infants with NF1. Error bars are ± 1 SE

**Fig. 2:**
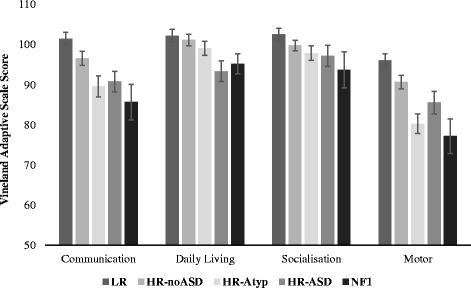
Average standard scores for the subscales of the Vineland Adaptive Behavior Scale from groups of infants with LR (low familial risk of ASD), HR-no ASD (high familial risk with later typical development), HR-Atyp (high familial risk with other atypical developmental profiles), HR-ASD (high familial risk with later ASD outcome), and infants with NF1. Error bars are ± 1 SE

There was a significant main effect of group on motor (Vineland Motor and Mullen Fine and Gross Motor) skills (*F*(12,624.7) = 6.36, *p* < .001, *η* ^2^ = 0.097; with age covaried *F*(12,622.043) = 5.86, *p* < .001, *η* ^2^ = 0.090). This reflected differences across Gross Motor (*F*(4,238) = 10.16, *p* < .001, *η* ^2^ = 0.15) and Fine Motor subscales of the Mullen (*F*(4,238) = 6.28, *p* < .001, *η* ^2^ = 0.096) and adaptive Motor skills on the VABS (*F*(4,238) = 9.28, *p* < .001, *η* ^2^ = 0.14; see Figs. 1 and 2). Bonferroni-corrected pairwise comparisons revealed that infants with NF1 showed significantly lower scores on Gross Motor skills on the Mullen relative to all four comparison groups (all *p*s < .01). Infants with NF1 showed significantly lower Fine Motor scores than the LR (*p* = 0.006) and HR-no ASD group (*p* = 0.009) but not the HR-Atyp (*p* = 0.5) or HR-ASD (*p* = 1) groups. On the VABS Motor domain, they showed lower scores than LR (*p* = 0.003) and marginally the HR-no ASD (*p* = .009) group but were comparable to the HR-Atyp (*p* = 1) and HR-ASD group (*p* = 0.93). The HR-ASD group also showed significantly lower scores than the LR group for the VABS motor domain (*p* = 0.033), Mullen fine motor skills (*p* = 0.012), and marginally Mullen gross motor scores (*p* = 0.099). Thus, both infants with NF1 and the HR-ASD group showed significantly poorer motor skills than low-risk infants.

#### Cognitive skills

There was a significant main effect of group on cognitive (Mullen visual reception and VABS Daily Living) skills (*F*(8,474) = 2.99, *p* = 0.003, *η* ^2^ = 0.048; with age covaried (*F*(8,472) = 3.08, *p* = 0.002, *η* ^2^ = 0.05). This group effect was seen for both Visual Reception (*F*(4,238) = 4.22, *p* = 0.003, *η* ^2^ = 0.066) and Daily Living scales (*F*(4,238) = 2.78, *p* = 0.028, *η* ^2^ = 0.045; see Figs. 1 and 2). Bonferroni-corrected pairwise comparisons revealed that infants with NF1 showed significantly lower Visual Reception scores than the LR group (*p* = 0.022), marginally lower than the HR-no ASD group (*p* = 0.11) but not the HR-Atyp (*p* = 1) or HR-ASD (*p* = 1) groups. Daily Living Skills were comparable to all other groups (*p* = 1). The HR-ASD group showed significantly lower scores for Daily Living Skills than the LR group (*p* = 0.033); this was not the case for Visual Reception (*p* = 0.3). Inspection of the means in Fig. 2 indicates that infants with NF1 showed comparable values to the HR-ASD group; thus, the absence of clear group differences may be related to the smaller size of that group.

#### Language

There was a significant main effect of group on language (Mullen Expressive and Receptive Language and VABS Communication) skills (*F*(12,624.7) = 5.25, *p* < 0.001, *η* ^2^ = 0.081; with age covaried (*F*(12,622.043) = 5.82, *p* < 0.001, *η* ^2^ = 0.090). This reflected differences across Receptive Language (*F*(4,238)= 7.42, *p* < 0.001, *η* ^2^ = 0.11), Expressive Language (*F*(4,238) = 6.03, *p* < 0.001, *η* ^2^ = 0.09), and adaptive Communication (*F*(4,238) = 6.02, *p* < 0.001, *η* ^2^ = 0.092; see Figs. 1 and 2). Bonferroni-corrected pairwise comparisons revealed that infants with NF1 showed significantly lower scores for Receptive Language than low-risk infants (*p* = 0.001) and the HR-TD (*p* < 0.001) and HR-Atyp (*p* = 0.007) groups but not the HR-ASD (*p* = 0.17) groups. For expressive language, infants with NF1 showed significantly lower scores than all other groups (*p*s ≤ 0.001). For Vineland Communication scores, the NF1 group showed significantly lower scores than LR infants only (*p* = 0.025; other groups *p*s > 0.3). The HR-ASD group showed significantly lower scores than the HR-no ASD group for receptive language (*p* = 0.007) than the LR group for VABS Communication (*p* = 0.025); other comparisons were not significant (*p*s > 0.3). Thus, both infants with NF1 and the HR-ASD group showed lower communication scores than LR infants.

#### Social functioning

**Fig. 3:**
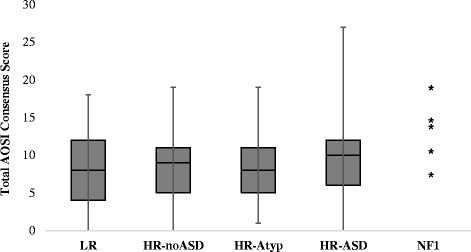
Box-and-whisker plots showing the distribution of total scores on the Autism Observation Scale for Infants (mean, lower and upper quartile, and whiskers show full range). No statistical comparisons were carried out as the sample size for NF1 was too small. Asterisks (*) depict the individual scores from the NF1 sample. Of note, this task was added to the protocol later in the study and so was only completed by five infants

There were no significant group differences in Vineland socialisation scores (*F*(4,239) = 2.063, *p* = 0.086, *η* ^2^ *=* 0.033). We did not compute statistical comparisons for the AOSI, since only five infants with NF1 completed it, but the distribution of scores within the NF1 group appeared similar to those for the HR-ASD group (Fig. 3).

#### Temperament

There were no significant group differences on the IBQ (*F*(12,622.043) = 1.25, *p* = 0.24, *η* ^2^ *=* 0.021).

## Discussion

We present the first developmental profiles of infants with NF1 and compare them to a large cohort of infants at familial risk with later ASD. Our initial report was designed to address two questions. First, what are the developmental challenges experienced by infants with NF1? Second, how do those challenges compare to infants with other familial routes to ASD? Findings reveal developmental delays across multiple domains that show some intriguing similarities to the pattern of difficulties seen in infants with familial risk routes to ASD.

### Developmental difficulties experienced by infants with NF1

Infants with NF1 showed broad developmental difficulties across a number of cognitive and motor domains. The most consistently affected areas were motor and communication skills, for which delays relative to low-risk infants were observed across both the Mullen and the Vineland. In addition, seven of the ten infants showed atypical scores on at least one domain of sensory responsivity, though the nature of the atypicality was heterogeneous. Similar developmental problems are observed in other genetic disorders linked to ASD, like Fragile X (FXS). For example, Hogan and colleagues report a case series of eight infants with FXS followed from 9 to 24 months of age, half of whom later met criteria for ASD [43]. Many infants showed relatively high levels of emergent autism-like behaviours on the Autism Observational Scale for Infants (AOSI), particularly those with a later outcome of autism. By the end of the first year, cognitive and adaptive deficits were seen in almost all infants and did not appear to differ by autism diagnosis. Thus, early developmental delays may be a common feature in infants with genetic syndromes linked to ASD.

Recent research has shown that the NF1 gene mutation confers a general vulnerability for cognitive difficulties in the preschool period. A study of 40 children with NF1 aged 3–6 years found weaker cognitive abilities on all subscales of the Differential Ability Scales compared to controls matched for age and socioeconomic status [47]. At least a third of preschool children with NF1 have difficulties with expressive and receptive language skills as well as phonological awareness [48]. A cross-sectional study of 39 toddlers with NF1 aged 21–30 months assessed using Bayley Scales of Infant Development, Wechsler preschool scale of intelligence, and parental measures of behaviour found poorer cognitive, motor, and language development in the NF1 group compared to age-matched controls [49]. Cognitive development was in the low to average range, 1 SD lower than controls, with below-average motor development in a third of the cohort. Further, parental responses indicated delayed receptive and expressive language development in over 70% of the NF1 cohort. Our current data indicates that these delays observed in preschoolers are present from at least 10 months of age and appear to be more pronounced in infancy. Longitudinal follow-up of our current cohort will be required to determine whether delays partially resolve over time.

Clinical information suggests low rates of identification of other co-occurring neurodevelopmental conditions within children with NF1, with substantial ‘diagnostic overshadowing’ in ascribing cognitive and behavioural problems to the NF1 diagnosis alone [25]. Diagnostic delays limit effectiveness of interventions, with life-long impacts on social and occupational functioning [50, 51]. There is an urgent need to develop early assessment and intervention approaches for ASD in NF1 that could significantly improve developmental outcomes for children. Our work indicates that developmental delays are apparent from at least 10 months of age, and so, early intervention may be particularly critical. Early interventions are not routinely provided; current guidelines recommend developmental assessment in order to support additional needs on starting school [52].

### NF1 and familial routes to ASD

Some elements of the developmental profile of infants with NF1 were similar to those seen in infants with familial routes to ASD. On the Vineland Adaptive Behavior Scales, socialisation profiles did not significantly differ between infants with NF1, infants with later ASD from familial risk cohorts, and low-risk infants. Further, infants with NF1 were rated as having relatively typical profiles of social engagement during the lab visit. Our rating scale was adapted from one used within infants with later ASD [46], and here too, infants were rated as relatively engaged at this age, with scores declining across the second year. It would be important to see whether infants with NF1 display the same profile of emerging social difficulties with age. At the group level on the Vineland, children with later ASD from our comparison samples showed the worst performance in motor skills and the best in socialisation and daily living, with communication skills at an intermediate level; the group means for children with NF1 show the same pattern albeit with more pronounced motor difficulties (see also [8] for no differences in socialisation at 12 months in infants at high familial risk for ASD). Longitudinal follow-up will be required to determine whether a similar profile is seen in the subgroup of infants with NF1 who develop later ASD. Nonetheless, our work suggests that relatively unaffected social functioning at the behavioural level at 10 months may be a shared phenotype between infants with both genetic and familial routes to ASD.

At the group level, developmental difficulties in infants with NF1 were generally more pronounced across all domains than in our comparison samples at familial risk of ASD, including those who went on to have an ASD outcome. This may be associated with the generally low likelihood of intellectual disability in samples of infants at familial risk relative to the broader population of children with ASD [2]. Interestingly, cognitive outcomes for children with NF1 tend to be better than for other neurodevelopmental conditions, so it will be important to determine whether over time many children catch up (to a degree) with their peers. Nonetheless, reports that the earliest behavioural signs related to later ASD are in motor skill (e.g. head lag) and sensory functioning [8–10] is somewhat consistent with the motor delays and atypical sensory responsivity we observed in infants with NF1. One important next step is to understand whether these early motor delays have cascading effects on later functioning and whether they can be related to observations in animal models of NF1. Interestingly, there is preclinical evidence of a role of the NF1 gene in skeletal development and growth [53] and normal muscle function [54]; further work should establish whether this contributes to the delays in gross motor skills seen in our infant cohorts.

Our children with later ASD from the familial risk group showed comparable profiles on the Mullen and Vineland. In contrast, children with NF1 generally showed more impairment on the Mullen than the Vineland. Interpreting these effects is difficult because of differences in the way the measures are administered. Possibly, parents of children with NF1 are less likely to recognise developmental difficulties. Further, some infants with NF1 did not have older siblings (*n* = 4) and this may affect how parents judge their early development. Another potential limitation is that we made group-based comparisons to a historical cohort of data (in order to present data on autism outcome) collected by different teams at the same site. Any differences between measure administration by different examiners would affect Mullen scores but not the Vineland or IBQ, because identical parent report forms were used across cohorts. Taken together, the consistent identification of motor and communication delays across both the Mullen and Vineland scales (despite their different limitations) gives particular confidence to these findings.

### Clinical implications

This initial case series is too preliminary for any definitive clinical implication, but it should alert the clinical community. Identification and early surveillance will often concentrate initially on the clinical genetic and neurocutaneous aspects. Our findings indicate an important focus for early developmental assessment and appropriate remediation in newly diagnosed NF1 infants.

### Limitations and future directions

Data collection for our project is still ongoing, and we do not yet know which of the infants with NF1 will later meet criteria for ASD or show elements of the broader ASD phenotype. Outcomes for children with genetic syndromes are highly heterogeneous [21]. ASD is not present in every child with NF1; many also develop other co-occurring conditions like epilepsy, intellectual disability, or severe attention problems. Thus, longitudinal prospective studies that can tease out predictors of these different outcomes are required. However, studying children with NF1 as a group (rather than dividing the group into those with and without ASD outcome) is the closest comparison to animal modelling approaches (which typically contrast NF1 knock-outs with wildtype). Given the rarity of NF1, our sample size is also relatively small, consistent with other recent reports on infants with rare disorders [43], [55]. Although our recruitment methods were designed to increase the likelihood that our cases would be representative of the broader population with NF1 (by recruiting through all UK genetic clinics), this remains a potential limitation to the generalisability of our conclusions and indicates the importance of continuing to build larger samples in this field. Because there are no previous reports from prospective studies of infants with NF1, it is impossible to determine the extent to which our sample is representative of the broader population of infants with NF1 in terms of ability level and outcome. Planned longitudinal follow-up in toddlerhood will be necessary to address this question. One potential difference is that within the general population, 50% of cases of NF1 are familial and 50% are sporadic [35]. Within our sample, 8/10 cases were familial and two infants had a de novo mutation identified postnatally through clinical presentation (usually the presence of café au lait spots). This imbalance is because familial NF1 is detected much earlier in development (through cord blood testing). Consistent with previous reports in older children [26] there did not appear to be anything clearly different about these two infants, though further work with larger samples is required to investigate this question fully. Further, it is important to note that in the present study there was remarkable consistency in the domains most affected across individual infants. For example, motor skills were delayed in almost all infants. Our findings are consistent with the proposal that infants with genetic syndromes may show somewhat more consistent profiles than infants with other routes to ASD, providing one way to constrain heterogeneity. Further, the observation of motor delays supports preclinical observations of a critical role for NF1 in musculoskeletal development [54]. Other limitations were that our comparison infants were slightly younger than infants with NF1, but we controlled for age in all analyses. Further, we did not have contextual data on the ITSP and social engagement scales from other cohorts, and thus, the interpretability of these measures is limited to the qualitative case reports.

## Conclusions

At 10 months, infants with NF1 in the present study showed delays in motor and communication functioning, with milder difficulties in visual reception. Seven out of ten infants showed atypicality in at least one domain of sensory function. In contrast, temperament and social engagement appear relatively typical. This profile shares some similarity with infants from familial risk samples who develop ASD, where very early behavioural difficulties are typically observed in sensory and motor domains (rather than social functioning). Our work introduces a new route to establishing a translational developmental neuroscience of ASD. Prospective longitudinal studies of infants with neurofibromatosis hold great promise for illuminating the neurodevelopmental systems that mediate between genetic risk and later behavioural symptoms. Our findings reveal a distinct profile of early impairment that will be of substantial interest to work on animal models of ASD. Further, our work indicates the critical importance of careful developmental monitoring of infants with NF1. Early delays should be identified and appropriate intervention provided.

## Additional file

Additional file 1:
**SM1** - Social Engagment Scale; **SM2** - Clinical Assessment; **SM Table 1** - Participant descriptive data and the assessments carried out for each group; **SM Table 2** - Standardised scores from the Mullen Scale for infants with NF1 (cases 1-10), infants with later ASD, and typically developing controls; **SM Table 3** - Standardized scores for the subscales and a composite score from the Vineland Adaptive Behavior Scale; **SM Table 4** - Infant Behavior Questionnaire scores for 14 subscales and 3 domain scales; **SM Table 5** - Sensory Sensitivity rated through the Infant/Toddler Sensitivity Profile for infants with NF1; **SM Table 6** - Experimenter-rated Social Engagement scores for infants with NF1; **SM Table 7** - Scores on the individual AOSI items, separated by sensory-motor and social abilities for infants with NF1. (DOCX 233 kb)

## Abbreviations

AOSI: Autism Observational Scale for Infants
ASD: Autism spectrum disorder
IBQ: Infant Behavior Questionnaire Short Form
ITSP: Infant/Toddler Sensory Profile
Mullen: Mullen Scales of Early Learning
NF1: Neurofibromatosis type 1
Vineland: Vineland Adaptive Behavior Scales
