# Supplementary material for: Early development of infants with neurofibromatosis type 1: a case series
Source: Mol Autism. 2017 Nov 23;8:62. doi: 10.1186/s13229-017-0178-0 (PMC5701449; doi:10.1186/s13229-017-0178-0)
Supplement: Additional file 1: — SM1 - Social Engagment Scale; SM2 - Clinical Assessment; SM Table 1 - Participant descriptive data and the assessments carried out for each group; SM Table 2 - Standardised scores from the Mullen Scale for infants with NF1 (cases 1-10), infants with later ASD, and typically developing controls; SM Table 3 - Standardized scores for the subscales and a composite score from the Vineland Adaptive Behavior Scale; SM Table 4 - Infant Behavior Questionnaire scores for 14 subscales and 3 domain scales; SM Table 5 - Sensory Sensitivity rated through the Infant/Toddler Sensitivity Profile for infants with NF1; SM Table 6 - Experimenter-rated Social Engagement scores for infants with NF1; SM Table 7 - Scores on the individual AOSI items, separated by sensory-motor and social abilities for infants with NF1. (DOCX 233 kb) [file 13229_2017_178_MOESM1_ESM.docx]

**Supplementary Materials**

**
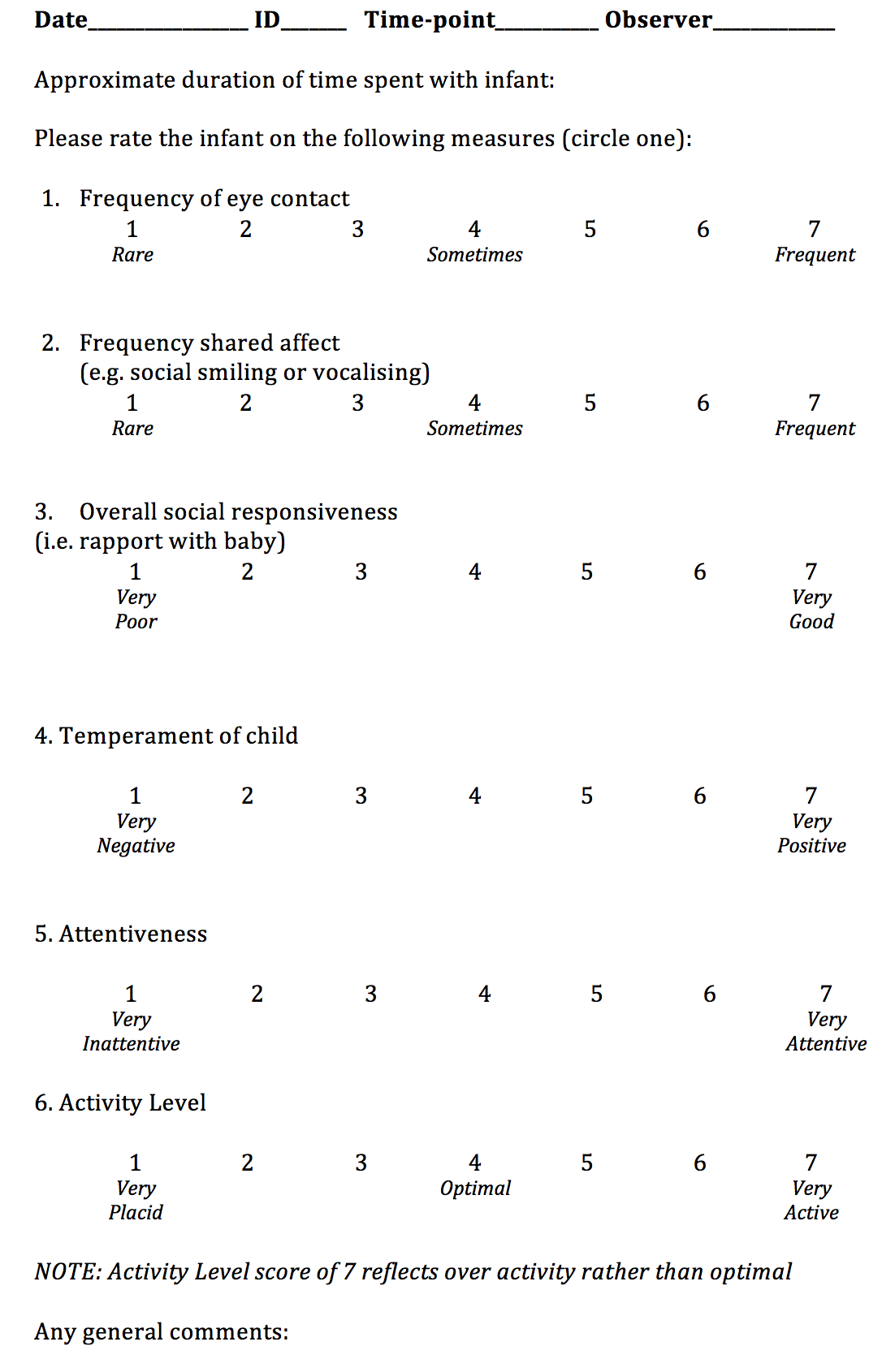
**SM1: Social Engagement Scale (consensus rated by experimenters post-visit)

***SM2: Clinical Assessment***

A battery of clinical research measures was administered to all children at 36 months (see Table 1):

the Autism Diagnostic Observation Schedule – Second Edition (ADOS-2;[54]), a standardised interaction observation assessment, was used to assess current symptoms of ASD (116 children were administered Module 2 and 20 children Module 1, ADOS not completed with 5 HR and 2 LR children). Calibrated Severity Scores for Social Affect, and Restricted and Repetitive Behaviors (RRB) were computed [55], which provide standardised autism severity measures, which account for differences in module administered, age and verbal ability. The Autism Diagnostic Interview – Revised (ADI-R; [56]), a structured parent interview, was also administered. Standard algorithm scores were computed for Reciprocal Social Interaction (Social), Communication, and Restricted, Repetitive and Stereotyped Behaviors and Interests (RRB). These assessments were conducted without blindness to risk-group status by or under the close supervision of clinical researchers (i.e., psychologists, speech therapists) with demonstrated research-level reliability. Total scores of the Social Communication Questionnaire (SCQ; [57]) were used as additional parent report measures of ASD symptoms. The early learning composite scaled score of the Mullen Scales of Early Learning (MSEL; [36]) were used to obtain a standardized measure of developmental abilities during testing and the 3 year visit.

Experienced clinicians (TC, GP, CC) reviewed information on ASD symptomatology (ADOS-2, ADI-R, SCQ), adaptive functioning (Vineland-II;[37]), and development (Mullen Scale of Early Learning, MSEL) for each HR and LR child to ascertain ASD diagnostic outcome according to DSM-5 (American Psychiatric Association, 2013).

*Table 1. Participant descriptive data and the assessments carried out for each group.*

| **Comparison** | **Group** | ***N* (males, females)** | **Chronological age**  **(months, days)** | **Assessments** |
| --- | --- | --- | --- | --- |
| *Case summary* | NF1 | 4,6 | 10m21d | AOSI, Mullen (M), Vineland Adaptive Behavior Scale (VABS), Infant Behavior Questionnaire Short Form(IBQ), Infant/Toddler Sensory Profile (ITSP), Social Engagement (SE) |
| *Standardized assessment* | HR-ASD | 26, 8 | 8m7d | AOSI, Mullen (M), Vineland Adaptive Behavior Scale (VABS), Infant Behavior Questionnaire Short Form (IBQ) |
|  | HR-noASD | 36, 53 | 8m17d |  |
|  | HR-Atyp | 23, 21 | 8m20d |  |
|  | LR | 35, 40 | 8m6d |  |

*Table 2. Standardised scores from the Mullen Scale for infants with NF1 (cases 1-10), infants with later ASD, and typically developing controls.*

| Case/Group | Gender/*N* |  |  | Mullen Scale of Early Learning | | |  |
| --- | --- | --- | --- | --- | --- | --- | --- |
|  |  | *Gross Motor* | *Fine Motor* | *Receptive Language* | *Expressive Language* | *Visual Reception* | *Composite* |
| **1** | M | **30** | *38* | 44 | *35* | 43 | *83* |
| **2** | M | 44 | 60 | **28** | 46 | 57 | 95 |
| **3** | M | **24** | *39* | *35* | **23** | 42 | *71* |
| **4** | M | **23** | 50 | *39* | **26** | 58 | 87 |
| **5** | F | *37* | *32* | *39* | 55 | **23** | *71* |
| **6** | F | **20** | 61 | *39* | 49 | 48 | 77 |
| **7** | F | *37* | 51 | **30** | *38* | 58 | 89 |
| **8** | F | **20** | **20** | **20** | **20** | *38* | **55** |
| **9** | F | **24** | 50 | *40* | **20** | 42 | *77* |
| **10** | F | **24** | *33* | **28** | *36* | 42 | *71* |
| **HR-ASD** | *n=33* | 44.18; 11.48; (20-72) | 49.33; 13.03; (26-80) | 42.85; 12.67; (20-70) | 50.06; 11.92; (26-76) | 51.45; 10.51; (32-80) | 97.12; 17.44; (64-143) |
| **HR-Atyp** | *n=43* | 44.91; 12.63; (20-65) | 51.77; 12.70; (20-80) | 46.16; 8.64; (23-61) | 51.14; 11.05; (26-76) | 50.23; 11.34; (28-75) | 99.81; 13.41; (74-130) |
| **HR-noASD** | *n=89* | 47.45; 10.50; (20-77) | 57.00; 12.56; (20-80) | 49.89; 10.04; (22-68) | 50.53; 9.98; (33-76) | 54.60; 11.76; (20-75) | 106.21; 15.49; (66-135) |
| **LR** | *n=73* | 50.10; 8.58; (30-68) | 58.03; 10.19; (27-80) | 47.37; 9.28; (26-70) | 51.93; 9.18; (33-76) | 56.26; 9.83; (32, 80) | 106.90; 12.13; (77-140) |

*Note*. Group averages of the standardised scores are provided for HR-ASD, HR-noASD, and LR (Mean; Standard Deviation; Range). Composite score distribution Mean of 100 and Standard Deviation of 15; while the subscales have a distribution Mean of 50 with Standard Deviation of 10).

Scores classified as ‘Very Low’ are depicted in **bold**; ‘Below Average’ in *italics*; and ‘Above Average’ performance scores are underlined.

*Table 3. Standardized scores for the subscales and a composite score from the Vineland Adaptive Behavior Scale.*

| Case/Group | Gender/*N* | Vineland Adaptive Behavior Scales | | | | |
| --- | --- | --- | --- | --- | --- | --- |
|  |  | *Communication* | *Daily Living Skills* | *Socialisation* | *Motor Skills* | *Composite* |
| **1** | M | *74* | 100 | 100 | **66** | *82* |
| **2** | M | 96 | 94 | 109 | *76* | 92 |
| **3** | M | 106 | 106 | 109 | 103 | 107 |
| **4** | M | 89 | 94 | 91 | *76* | *85* |
| **5** | F | **70** | 100 | *81* | **66** | *76* |
| **6** | F | *82* | *82* | 91 | **69** | *78* |
| **7** | F | 93 | 106 | 94 | *80* | 91 |
| **8** | F | **62** | 88 | **62** | **58** | **64** |
| **9** | F | 100 | 94 | 106 | 87 | 96 |
| **10** | F | *85* | 88 | 94 | 91 | 87 |
| **HR-ASD** | *n=34* | 90.82; 15.13; (40-112) | 93.44; 15.24; (58-117) | 97.15; 15.35; (53-126) | 85.62; 16.39; (54-119) | 90.32; 13.80; (49-113) |
| **HR-Atyp** | *n=43* | 89.60; 17.14; (42-118) | 99.12; 11.57; (72-117) | 97.86; 11.53; (73-126) | 80.26; 15.81; (46-113) | 90.00; 11.55; (68-114) |
| **HR-noASD** | *n=86* | 96.62; 15.84; (55-143) | 101.23; 13.38; (54-143) | 99.76; 11.96; (70-152) | 90.65; 15.99; (56-144) | 96.23; 13.06; (66-150) |
| **LR** | *n=71* | 101.48; 13.72; (66-137) | 102.24; 13.72; (54-136) | 102.63; 12.75; (77-143) | 96.08; 14.00; (73-137) | 100.42; 12.38; (66-144) |

*Note*. Group averages of the standardised scores are provided for HR-ASD, HR-noASD, and LR (Mean; Standard Deviation; Range). Composite score distribution Mean of 100 and Standard Deviation of 15. Scores classified as ‘Low’ (<=-2SD) are depicted in **bold**; ‘Moderately Low’ (<=-1SD) in *italics*.

*Table 4. Infant Behavior Questionnaire scores for 14 subscales and 3 domain scales*

| Case/  Group | Gender/*N* | IBQ Domain Scales | | | |
| --- | --- | --- | --- | --- | --- |
|  |  | ***Surgency*** | ***Negative Affect*** | ***Effortful Control*** |  |
| **1** | M | 4.56 | 3.66 | 5.10 |  |
| **2** | M | 5.08 | 4.01 | *3.79* |  |
| **3** | M | 5.42 | 3.08 | *4.21* |  |
| **4** | M | 4.65 | **4.70** | 4.52 |  |
| **5** | F | 4.69 | 3.43 | *4.21* |  |
| **6** | F | 4.43 | 2.49 | 5.17 |  |
| **7** | F | *4.03* | **5.43** | *4.23* |  |
| **8** | F | ***3.48*** | 3.24 | *4.2* |  |
| **9** | F | *4.17* | *2.12* | 6.18 |  |
| **10** | F | 6.43 | **4.81** | ***1.81*** |  |
| **HR-ASD** | *n=34* | 4.86; 0.69; (3.08-6.16) | 3.52; 1.01; (1.77-6.18) | 4.77; 0.67; (3.66-6.55) |  |
| **HR-Atyp** | *n=41* | 4.70; 0.68; (2.66-6.4) | 3.50; 0.81; (2.09-5.66) | 4.71; 0.64; (2.69-5.90) |  |
| **HR-noASD** | *n=87* | 4.74; 0.71; (3.04-6.36) | 3.42; 0.71; (1.73-5.43) | 4.66; 0.67; (2.87-6.17) |  |
| **LR** | *n=71* | 4.80; 0.56; (3.85-6.24) | 3.22; 0.68; (1.72-5.2) | 4.86; 0.55; (3.61-6.07) |  |

*Note.* Group averages of the standardised scores are provided for HR-ASD, HR-noASD, and LR (Mean; Standard Deviation; Range).

For individual case series, domain scores were compared to the low-risk control group: Underline – one standard deviation above the mean; **Bold underline** – two standard deviations above the mean; *italic* – one standard deviation below the mean; ***bold italic*** – two standard deviations below the mean.

*Table 5. Sensory Sensitivity rated through the Infant/Toddler Sensitivity Profile for infants with NF1.*

| Case/Group | Gender | Sensory processing | | | | | Quadrant Summary | | |
| --- | --- | --- | --- | --- | --- | --- | --- | --- | --- |
|  |  | *Auditory* | *Visual* | *Tactile* | *Vestibular* | *Oral Sensory* | *Low Registration* | *Sensation Seeking* | *Low Threshold* |
| **1** | M | **27** | 18 | 46 | **10** | **16** | **29** | 27 | **70** |
| **2** | M | *44* | 23 | 58 | 19 | 22 | 52 | ***49*** | 104 |
| **3** | M | 35 | 19 | 55 | 19 | 26 | ***46*** | 23 | 97 |
| **4** | M | 42 | 23 | 55 | 16 | 26 | 49 | 31 | 97 |
| **5** | F | **23** | 17 | 42 | 20 | 19 | **29** | 20 | 85 |
| **6** | F | 40 | 25 | 54 | 23 | 22 | 47 | 32 | 95 |
| **7** | F | **29** | 19 | 57 | 18 | 22 | 44 | 23 | 90 |
| **8** | F | **30** | 18 | 50 | **13** | **16** | **35** | 20 | 81 |
| **9** | F | 41 | 19 | 56 | 19 | 22 | *55* | 15 | 102 |
| **10** | F | 38 | 23 | 59 | 21 | 27 | 51 | 26 | 106 |

*Note.* Quadrant and sensory processing scores calculated according to [44] and rated relative to performance of children 7-12 months with typical development. Underline – probably more than others, **Bold underline** – definitely more than others, *Italics* – probably less than others, ***Bold Italics*** – definitely less than others.

*Table 6. Experimenter-rated Social Engagement scores for infants with NF1.*

| Case/Group | Gender | Social Engagement | | | | | |
| --- | --- | --- | --- | --- | --- | --- | --- |
|  |  | *Eye Contact* | *Shared Affect* | *Social Responsiveness* | *Temperament* | *Attentiveness* | *Activity Level* |
| **1** | M | 4 | 2 | 4 | 3 | 4 | 5 |
| **2** | M | 7 | 7 | 6 | 5 | 5 | 7 |
| **3** | M | 4 | 3 | 4 | 5 | 5 | 5 |
| **4** | M | 7 | 6 | 6 | 6 | 5 | 3 |
| **5** | F | 5 | 5 | 4 | 5 | 3 | 4 |
| **6** | F | 6 | 4 | 5 | 6 | 2 | 5 |
| **7** | F | 7 | 4 | 5 | 2 | 4 | 5 |
| **8** | F | 4 | 2 | 3 | 3 | 3 | 2 |
| **9** | F | 7 | 5 | 5 | 6 | 4 | 4 |
| **10** | F | 5 | 3 | 3 | 3 | 5 | 4 |

*Note.* Consensus scored by experimenters on the day of testing. Eye-contact and Shared Affect rated between rare (1) and 7(frequent); Social Responsiveness, Temperament, and attentiveness rated between very poor/ negative (1) and very good/positive (7); Activity Level between very placid (1), optimal (4), or very active (7).

*Table 7. Scores on the individual AOSI items, separated by sensory-motor and social abilities for infants with NF1.*

| Case | Gender | Sensory-Motor | | | | | | | | | |
| --- | --- | --- | --- | --- | --- | --- | --- | --- | --- | --- | --- |
|  |  | *Visual tracking* | *Disengagement of attention* | *Anticipatory response* | *Coordination of gaze and action* | *Reactivity* | *Transitions* | *Motor control and behavior* | *Atypical motor behaviors* | | *Atypical sensory behaviors* |
| **3** | M | 0 | 0 | 0 | 1 | 0 | 0 | 2 | 0 | | 0 |
| **5** | F | 0 | 0 | 0 | 0 | 1 | 1 | 2 | 2 | | 2 |
| **7** | F | 0 | 0 | 0 | 0 | 1 | 0 | 0 | 0 | | 0 |
| **8** | F | 0 | 2 | 0 | 0 | 1 | 1 | 2 | 2 | | 2 |
| **10** | F | 0 | 0 | 0 | 0 | 2 | 0 | 1 | 2 | | 2 |
|  |  | Social | | | | | | | | | |
|  |  | *Orientation to name* | *Differential response to facial emotion* | *Imitation of actions* | *Social babbling* | *Eye-contact* | *Reciprocal social smile* | *Social interest and shared affect* | |  |  |
| **3** | M | 2 | 0 | 0 | 3 | 2 | 2 | 2 | |  |  |
| **5** | F | 0 | 0 | 0 | 0 | 0 | 2 | 1 | |  |  |
| **7** | F | 1 | 1 | 0 | 1 | 0 | 2 | 1 | |  |  |
| **8** | F | 0 | 1 | 0 | 2 | 2 | 3 | 1 | |  |  |
| **10** | F | 0 | 2 | 0 | 3 | 0 | 2 | 1 | |  |  |

*Note.* AOSI is scored from 0 to 2 or 3, where 0 denotes typical function, and scores 1-3 are rated for severity of impairment; where a rating of 3 is a complete lack of behavior. Eye-contact, atypical motor and sensory behaviors are the three exceptions, and the choice is confined to 0 (typical) and 2 (atypical) [40].
